# The interaction between cytosine methylation and processes of DNA replication and repair shape the mutational landscape of cancer genomes

**DOI:** 10.1093/nar/gkx463

**Published:** 2017-05-22

**Authors:** Rebecca C. Poulos, Jake Olivier, Jason W.H. Wong

**Affiliations:** 1 Prince of Wales Clinical School and Lowy Cancer Research Centre, UNSW Sydney, NSW 2052, Australia; 2 School of Mathematics and Statistics, The Red Centre, UNSW Sydney, NSW 2052, Australia

## Abstract

Methylated cytosines (5mCs) are frequently mutated in the genome. However, no studies have yet comprehensively analysed mutation–methylation associations across cancer types. Here we analyse 916 cancer genomes, together with tissue type-specific methylation and replication timing data. We describe a strong mutation–methylation association across colorectal cancer subtypes, most interestingly in samples with microsatellite instability (MSI) or *Polymerase epsilon (POLE)* exonuclease domain mutations. By analysing genomic regions with differential mismatch repair (MMR) efficiency, we suggest a possible role for MMR in the correction of 5mC deamination events, potentially accounting for the high rate of 5mC mutation accumulation in MSI tumours. Additionally, we propose that mutant POLE asserts a mutator phenotype specifically at 5mCs, and we find coding mutation hotspots in *POLE-*mutant cancers at highly-methylated CpGs in the tumour-suppressor genes *APC* and *TP53*. Finally, using multivariable regression models, we demonstrate that different cancers exhibit distinct mutation–methylation associations, with DNA repair influencing such associations in certain cancer genomes. Taken together, we find differential associations with methylation that are vital for accurately predicting expected mutation loads across cancer types. Our findings reveal links between methylation and common mutation and repair processes, with these mechanisms defining a key part of the mutational landscape of cancer genomes.

## INTRODUCTION

Cancer develops as somatic mutations accumulate in cells, with certain driver mutations conferring a growth advantage to a sub-population (1). In some cancers, mutations develop primarily from exposure to exogenous mutagens such as ultraviolet (UV) light or cigarette smoke, while in other cancer types, most mutations accumulate after a cell develops defective replication or repair mechanisms (2). Mutation rates vary throughout the cancer genome due to factors such as trinucleotide composition (3), transcription factor binding (4,5), chromatin organisation (6), replication timing and mismatch repair (MMR) efficiency (7). However, the origin of many mutations within cancer cells still remains unknown (3).

DNA methylation is an epigenetic mark, most commonly occurring in the genome at sites of CpG dinucleotides (8). Cytosine methylation involves the covalent attachment of a methyl group to the fifth atom of the carbon ring of a cytosine, forming molecules known as 5-methylcytosine (5mC) (9). Methylation has important functions within a cell, influencing development (10), gene expression and silencing (11), as well as being implicated in carcinogenesis (12).

Despite its crucial role in cellular function however, CpG methylation can also be somewhat mutagenic, with methylated cytosines being approximately fivefold more likely to undergo spontaneous deamination (loss of an amine group) than unmethylated cytosines (13). 5mC deamination yields thymine, leading to a G•T mismatch in DNA which can be recognized by thymine DNA glycosylases and repaired through the base excision repair (BER) pathway (14,15). However, if a cell replicates before the mismatch can be repaired, a C>T mutation will become encoded into its genome. A mutation signature from cytosine deamination at CpG sites—signatures 1A and 1B from Alexandrov *et al*. (3)—has been identified in many cancer types, and is strongly correlated with age of diagnosis as, over time, more deamination events can occur and their mutagenic effects accumulate (16). Methylated CpG dinucleotides (mCpGs) have additionally been found to be highly mutated in non-cancer tissues, with mutation rates also correlating with increasing age (17).

The commonly accepted mechanism of mCpG mutation is that mutations accumulate solely due to random spontaneous deamination of 5mC. However, other processes have also been associated with 5mC mutation or deamination, including exposure to UV light or to cigarette smoke (18). In addition, understanding the repair of G•T mismatches is crucial for determining how mutations at sites of 5mC accumulate within the genome (19). In this study, we analyse the association between methylation and mutation in 61 whole-genome sequenced (WGS) colorectal cancers, together with an additional 855 whole-genomes across 11 cancer types. We describe the association in detail within colorectal cancer subtypes, positing a potential role for MMR in the correction of deaminated 5mCs, and suggesting that *Polymerase epsilon* (*POLE*) exonuclease domain mutation increases mutagenesis specifically at 5mCs. We further define the influence of methylation and replication timing on mutation accumulation and repair in cancer, describing distinct mutation–methylation associations in different cancer types, and pinpointing nucleotide excision repair (NER) to be pertinent to mutation profiles at 5mCs in certain cancer genomes.

## MATERIALS AND METHODS

### Somatic mutations and sample classification

Raw data and somatic mutation calls were obtained from The Cancer Genome Atlas (TCGA) Cancer Genomics Hub (CGhub) (20), International Cancer Genome Consortium (ICGC) (21), or previously published datasets (3,22). Data sources and cancer samples for each cancer type are listed in Supplementary Table S1A and Supplementary Table S1B, with data processing as described (4). In brief, for cancer data obtained from TCGA, mutations were called from BAM files using Strelka (23), with only mutations listed as ‘PASS’ selected for analysis. For cancers with data obtained from ICGC, ‘single base substitutions’ were obtained directly from the ICGC data portal (release 16). Somatic mutations from Alexandrov *et al*. (3) were obtained from ftp://ftp.sanger.ac.uk/pub/cancer/AlexandrovEtAl and mutations from Zheng *et al*. (22) were obtained from the database of Genotypes and Phenotypes (dbGap) (phs000830). Colorectal cancers with microsatellite instability (MSI) were selected if they were designated as MSI high (MSI-H) via annotations from TCGA. Tumours with *POLE* exonuclease domain mutations (*POLE*-mutant tumours) were designated as such if they had both a somatic mutation in the exonuclease domain (amino acids 268–471) (24) and a Pearson's correlation >0.85 with signature 10 (3). See Supplementary Materials and Methods for further details of cancer subtype classifications.

### Methylation, replication timing and repair data

Methylation data from normal sigmoid colon tissue were downloaded from the Roadmap Epigenomics Atlas (25) (Gene Expression Omnibus [GEO]: GSM983645). These data were from whole genome bisulfite sequencing (WGBS) obtained as a wig file and converted to BED format using ‘convert2bed’. Methylation values and chromosome coordinates for individual nucleotides in each CpG were merged, taking the value for methylation as that from the cytosine of each CpG dinucleotide. This value was then used for all methylation calculations relating to colorectal cancer mutations throughout this study. Additional methylation datasets were obtained from the Roadmap Epigenomics Atlas (25) and analysed similarly. These datasets were matched to various cancer types and subtypes as listed in Supplementary Table S2, together with their GEO accession numbers. Normal tissue methylation data have been used for all analyses, which is a limitation of our study. However, while sample-matched methylation data are available for some samples, these bisulfite-based technologies are unable to distinguish between a bisulfite-converted cytosine to uracil change (read as C>T) at unmethylated cytosines and methylation-induced spontaneous mCpG deamination resulting in a C>T change, and hence these sample-matched data are inappropriate for use in this study.

Genome-wide replication timing datasets were downloaded from the UCSC Genome Browser (also available through GEO as GSE34399). GM12878 was the only lymphoblastoid cell-line used, to avoid biasing the sample through inclusion of multiple lymphoblastoid cell-lines, as previously described (7). The remaining datasets contained replication timing values for 11 cell-types. The genome was divided into megabase windows using BEDtools (26), with replication timing averaged across cell-types within these windows.

Excision repair sequencing (XR-seq) data for skin fibroblast cell line NHF1 (27) were obtained in Sequence Read Archive (SRA) format (GEO: GSE67941), and processed as previously described (28).

### Statistical analyses

Regression models and other statistical analyses were performed in R. For each cancer type or subtype, the binary logistic regression model predicting mutation probability incorporated methylation (with a quadratic term), replication timing and an interaction between methylation and replication timing, as shown below:

\begin{equation*}\log \left( {\frac{{{P_{mut}}}}{{1 {-} {P_{mut}}}}} \right) {=} {b_0} {+} {b_1}M {+} {b_2}{M^2} {+} {b_3}R {+} {b_4}\left( {M \times R} \right)\end{equation*}

where *P*_mut_ = probability of mutation; *M* = methylation; *R* = replication timing; *b*_0_, *b*_1_, *b*_2_, *b*_3_ and *b*_4_ represent constants estimated from logistic regression.

This model was selected for use as it significantly improved upon nested binary logistic regression models with fewer terms (data not shown). A significant improvement was determined by use of both a Likelihood Ratio Test (LRT; ‘lrtest’ function from the ‘lmtest’ package (29); model selected if LRT showed significant improvement by *P* < 0.05 at all steps between nested models) and the Akaike Information Criterion (AIC; model with smallest AIC was selected). Regression models were constructed using data for autosomes only. Mutations were considered a binary outcome, with each CpG designated as either never mutated in any sample, or mutated in at least one sample, within a given cancer type or subtype. The area under the curve (AUC) was calculated using the ROCR package (30). Equations predicted by the regression models, together with the predicted vertex and AUC from relevant nested models, are recorded in Table 1 and Supplementary Table S3. For further details of regression modelling or other statistical analyses, refer to Supplementary Materials and Methods.

**Table 1.** Regression equation from multivariable models predicting mutation probability across colorectal and squamous cell carcinoma subtypes, together with vertex and area under curve (AUC) predictions

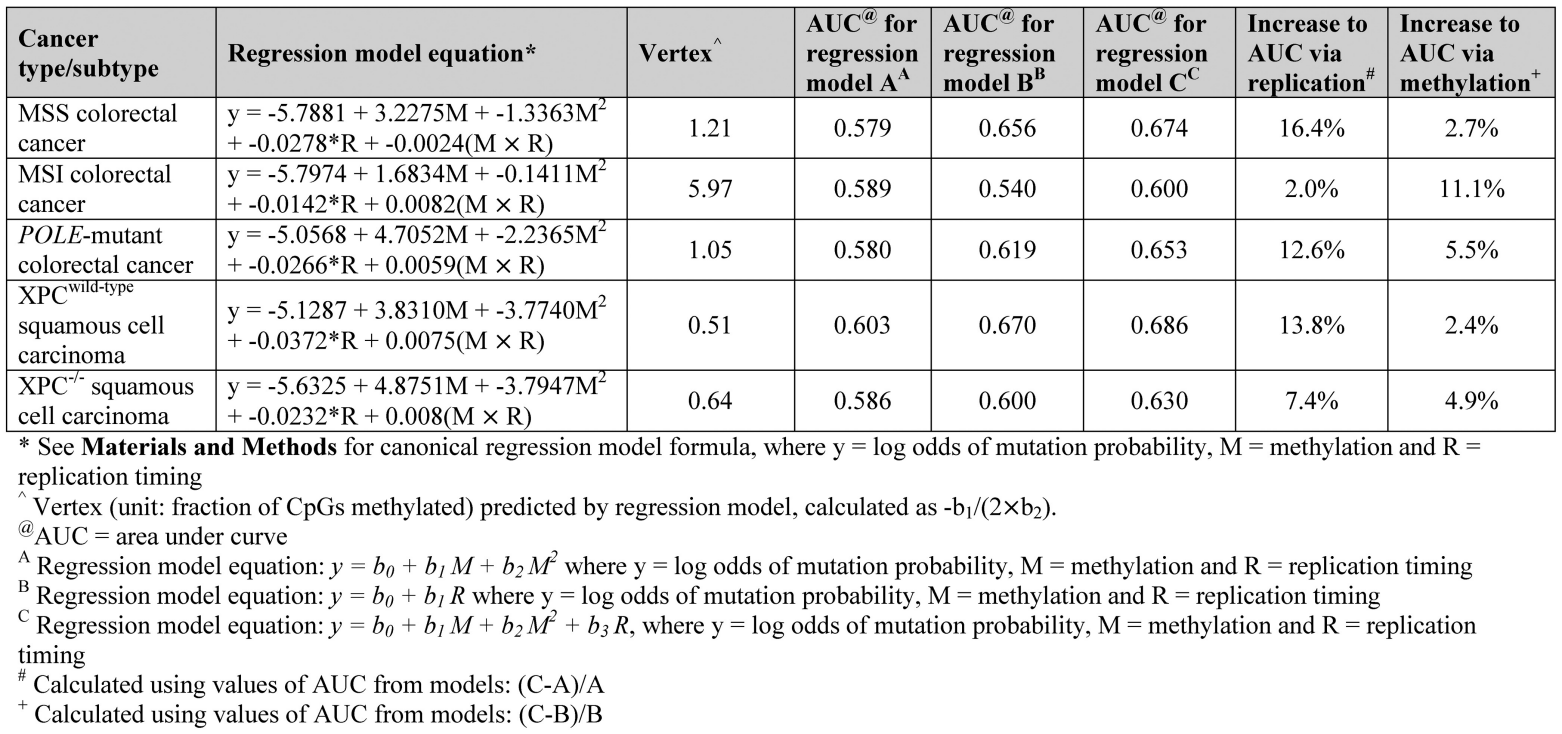

## RESULTS AND DISCUSSION

### Methylation and mutation associations in colorectal cancer

**Figure 1.**
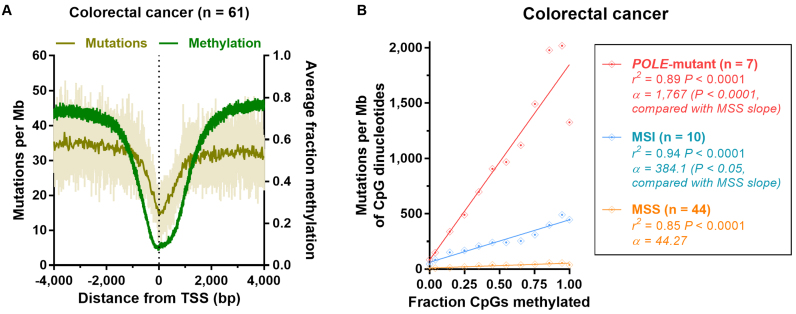
Association between mutation accumulation and methylation in colorectal cancer subtypes. (**A**) Colorectal cancer (*n* = 61) mutation profile and average methylation profile from normal colon whole genome bisulfite sequencing (WGBS) data, around transcription start sites (TSSs). Nucleotide-resolution mutation data (light beige), together with mutation data in 25 bp bins (dark beige) is shown. (**B**) Correlation between mutations per megabase (Mb) at CpG dinucleotides and fractions of CpGs methylated (using normal sigmoid colon tissue WGBS methylation data) across autosomes in *Polymerase epsilon* exonuclease domain mutation (*POLE*-mutant) colorectal cancers, those with microsatellite instability (MSI) or those that are microsatellite stable (MSS). Genome-wide data is binned for each colorectal cancer subtype (bins of 0.1 methylation), along with *r*^2^ and significance from Pearson's regression. The comparison of MSI and *POLE*-mutant slopes with MSS slopes was calculated by linear regression on binned data, with MSS as the reference factor.

Recent studies investigating the accumulation of somatic mutations in cancer have shown that mutations in many cancer types increase at promoters due to inhibition of NER at transcription factor bindings sites (4,5). Colon cancers have the lowest relative rate of mutations at promoters, attributable to the reduced importance of NER in the repair of mutations accumulating in such tissues (4). In this study, we have investigated the reduction of promoter mutations in colorectal cancer further. To do so, we constructed mutation profiles around transcription start sites (TSSs) using 61 WGS colorectal cancer samples from TCGA and observed a decrease in mutation load in the region immediately surrounding the TSS (Figure 1A). To understand this feature across colorectal cancer subtypes, we separated these colorectal cancer samples into those which were microsatellite stable (MSS), MSI or *POLE*-mutant. We found each of the subtypes to exhibit reduced mutation loads at the TSS, with more pronounced relative hypo-mutation in MSI and *POLE*-mutant samples (Supplementary Figure S1A).

As CpG methylation is typically lower at CpG Island (CGI)-associated promoter elements (31), we investigated methylation around the TSS using normal sigmoid colon WGBS data (25). We mapped average CpG methylation, observing a corresponding decrease in methylation in the region immediately surrounding the TSS (Figure 1A; see also Supplementary Figure S1B for DNase I hypersensitivity (DHS) and H3K4me3 profiles around the TSS—indicating promoter activity). Given this association, we correlated normal colon tissue methylation with mutations per megabase (Mb) of CpG dinucleotides across autosomes in colorectal cancer to determine whether more highly-methylated sites are more frequently mutated. We found there to be a significant association between mutation load and methylation (with methylation in bins containing increasing fractions of CpGs methylated) in each colorectal cancer subtype (*P* < 0.0001, Pearson's correlation; Figure 1B). Further, we observed significantly steeper slopes of association for *POLE*-mutant and MSI samples when compared with the slope for MSS samples [*P* < 0.0001 (*POLE*-mutant) and *P* < 0.05 (MSI), linear regression; Figure 1B]. We propose that the baseline-association observed in MSS samples represents endogenous mCpG deamination and repair rate in colon tissue. Therefore, this finding demonstrates that the increased CpG mutation loads of MSI and *POLE*-mutant colorectal cancers must be attributable either to methylation-associated mutagenesis or to methylation-associated repair deficiencies.

### Potential role for mismatch repair in the correction of 5mC deamination-induced mismatches

We find no evidence in the literature to suggest that loss of MMR increases the rate of spontaneous deamination at mCpG dinucleotides. We suggest therefore, that the difference that we have observed between the rate of mutation accumulation at mCpGs in MSS and MSI colorectal cancer genomes must instead be due to a methylation-associated repair deficiency.

**Figure 2.**
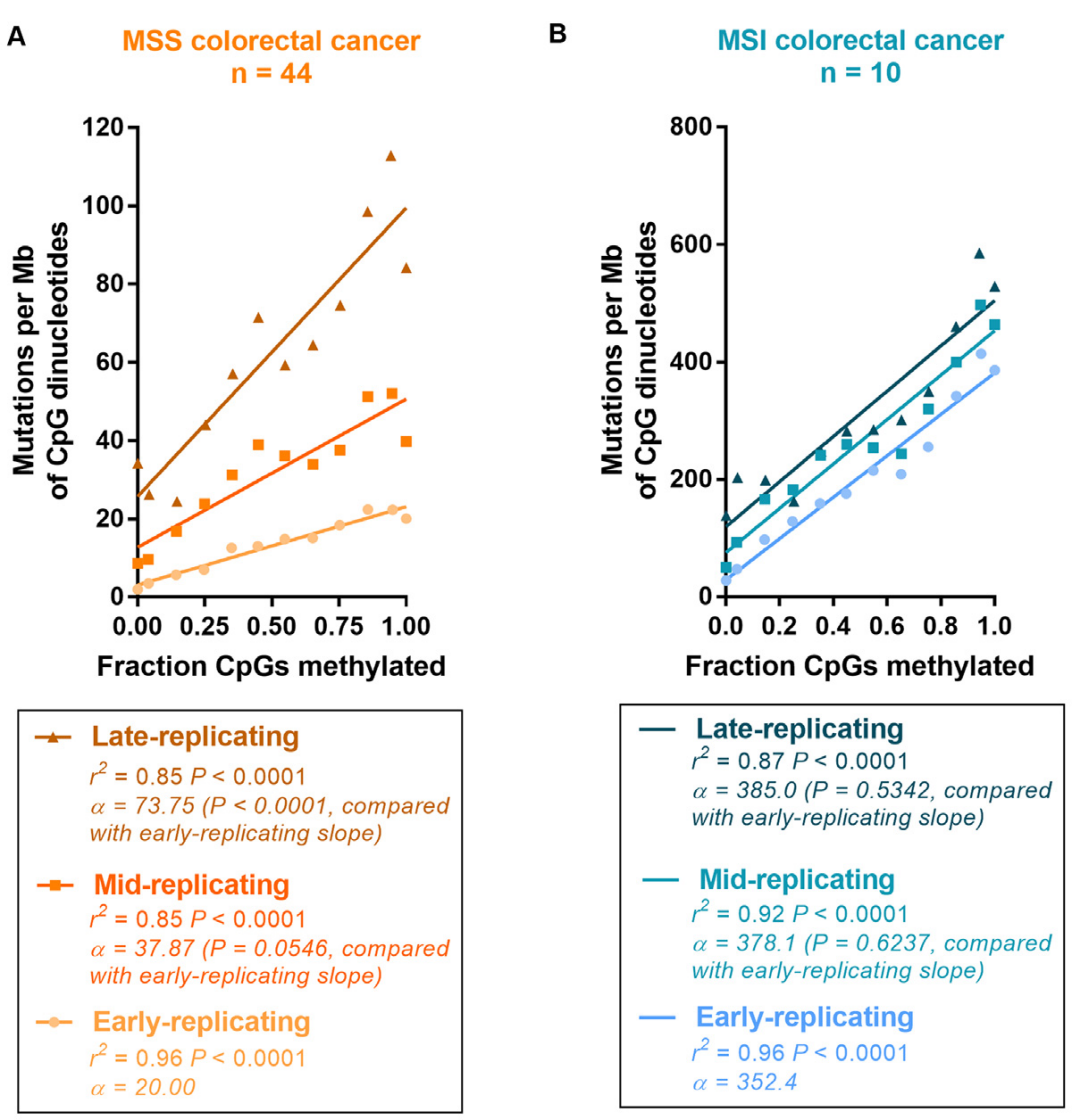
Association between mutation accumulation and methylation across changes in replication timing in colorectal cancers with differential mismatch repair. Correlation between mutations per megabase (Mb) at CpG dinucleotides and fractions of CpGs methylated (using normal sigmoid colon tissue WGBS methylation data) across autosomes in (**A**) microsatellite stable (MSS) colorectal cancers, and (**B**) colorectal cancers with microsatellite instability (MSI). Genome-wide data is binned for each colorectal cancer subtype (bins of 0.1 methylation), along with *r*^2^ and significance from Pearson's regression. The comparison of mid- and late-replicating slopes with early-replicating slopes was calculated by linear regression on binned data, with ‘early-replicating’ as the reference factor.

MMR efficiency differs with replication timing, as MMR is more active in early-replicating regions (7). Therefore, if MMR is involved to a significant extent in the repair of mCpG deamination-induced mismatches, mutations would accumulate at a greater rate at highly-methylated sites in later-replicating regions where MMR efficiency is poorer. We found this to be the case in MSS (Figure 2A) and *POLE*-mutant genomes (Supplementary Figure S2), both of which are MMR-proficient. In these genomes, we found the slope of the mutation–methylation association to be increased in mid- and late-replicating regions, when compared with early-replicating regions [MSS: *P* = 0.0546 (mid) and *P* < 0.0001 (late); *POLE*-mutant: *P* < 0.01 (mid) and *P* < 0.0001 (late), linear regression; Figure 2A and Supplementary Figure S2]. In contrast, in MSI cancers, where most mutations accumulate in the absence of MMR, we do not observe the effects of differing replication timing on the correction of mCpG deamination-induced mismatches. In these cancers, we found there to be no difference in the slope of mutation–methylation associations in mid- or late-replicating regions, when compared with early-replicating regions [*P* = 0.6237 (mid) and *P* = 0.5342 (late), linear regression; Figure 2B]. Taken together, these data would support a role for MMR in the repair of deamination-induced mismatches at mCpG dinucleotides.

It is worth noting however, that BER via *methyl-CpG binding domain 4 (MBD4)* and *thymine-DNA glycosylase (TDG)* is more commonly associated with the repair of 5mC deamination-induced G•T mismatches (14,15). Studies have shown that their impairment can cause increased transition mutations at mCpGs (32–34). In particular, *MBD4* has been found to be altered at high rates in MSI colorectal cancers due to MSI-induced mutations in polynucleotide tracts within the coding region of *MBD4* (35–37). Indeed, we found that 4/10 of our MSI samples harboured a truncation of MBD4. However, as the mutation–methylation association is no different between MSI samples with and without MBD4 truncation, we find it unlikely that MBD4 inactivation is solely responsible for the increased rate of mutation accumulation that we observe at 5mCs in MSI cancers (see Supplementary Data Note). Alternative explanations are that the samples without MBD4 truncations harbor other defects in BER that we have not detected, or that the loss of MMR also simultaneously leads to the impairment of BER activity. To further elucidate whether MMR plays a direct role in repairing errors at mCpG dinucleotides, or somehow indirectly impacts the correction of errors at mCpG dinucleotides, would require further research.

### Mutagenesis at 5mC nucleotides in *POLE*-mutant colorectal cancers

**Figure 3.**
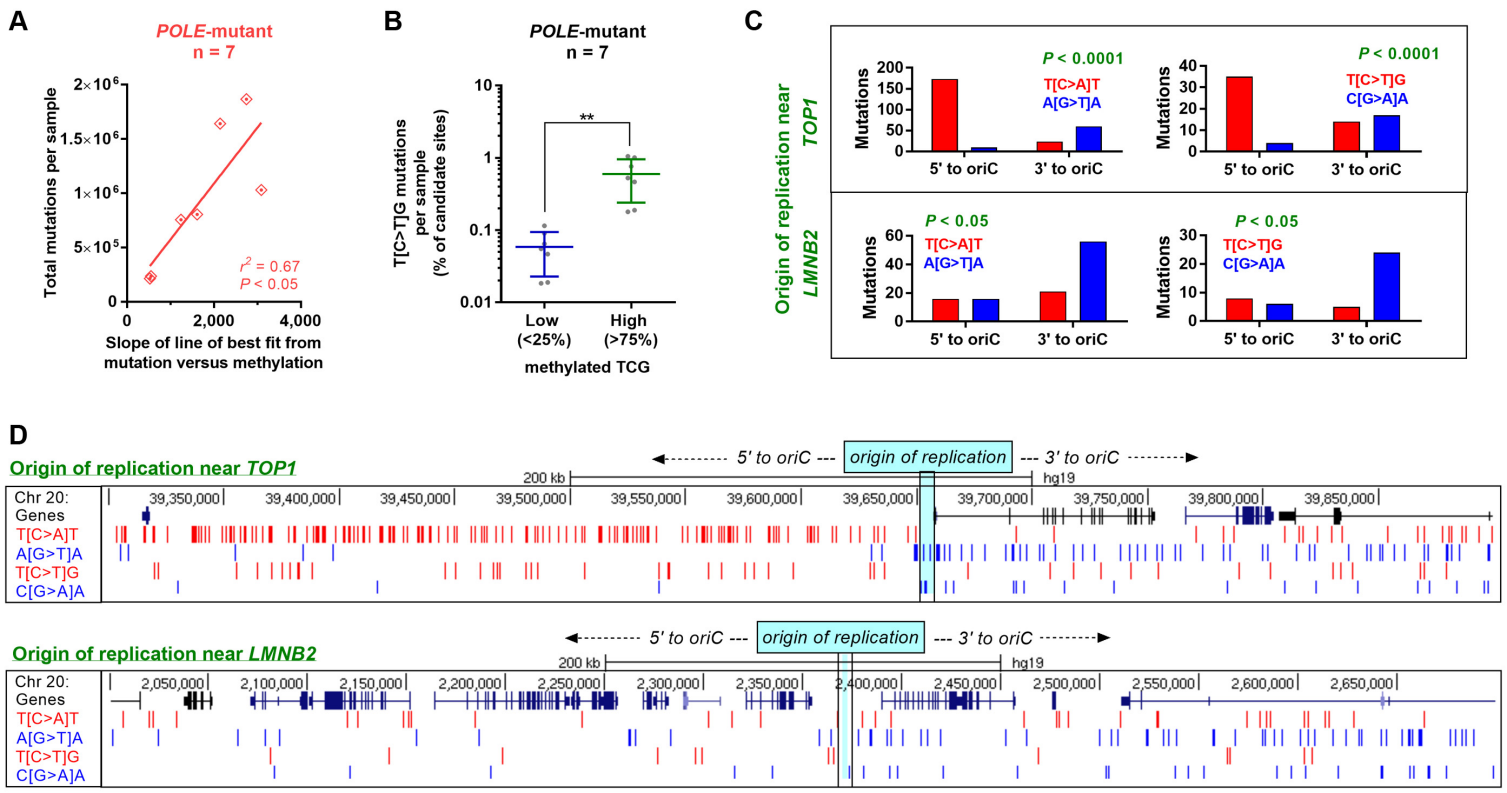
Methylation-associated mutations in *POLE*-mutant colorectal cancers. (**A**) Correlation of total mutations per *Polymerase epsilon* exonuclease domain mutant (*POLE*-mutant) colorectal cancer sample, with the slope of line of best fit from the mutation–methylation association at Supplementary Figure S3. *r*^2^ and significance is by Pearson's correlation. (**B**) Percentage of candidate sites which harbour C>T mutations in a TCG context per sample for low (<25%) and high (>75%) methylated CpGs (using normal colon tissue methylation data) per *POLE*-mutant colorectal cancer sample. Mean and standard deviation are shown; significance is by unpaired *t*-test where **P < 0.01. (**C**) Strand-specificity of T[C>A]T (left) and T[C>T]G (right) mutations in the regions 5΄ and 3΄ to origins of replication (oriC) near *TOP1* (top) and *LMNB2* (bottom). Significance is by Fisher's exact test. (**D**) Excerpt from the UCSC genome browser, depicting strand specificity of T[C>A]T and T[C>T]G mutations 5΄ and 3΄ to the oriC near *TOP1* (top) and *LMNB2* (bottom).

Focusing next on the association between methylation and mutation accumulation in *POLE*-mutant tumours, we computed the correlation coefficient between CpG mutations and methylation for individual *POLE*-mutant colorectal cancer samples. We found that the slope of the line of best fit from binned data comparing CpG mutations to fractions of CpGs methylated, ranged from 521.7 to 3090 (Supplementary Figure S3), with a significant positive correlation between the slope of each line and the total number of mutations in each *POLE*-mutant sample (*r*^2^ = 0.67 *P* < 0.05, Pearson's correlation; Figure 3A), and confirming our observation that much of the increased mutagenesis at CpGs in *POLE*-mutant cancers is methylation-associated (see Figure 1B). *POLE*-mutant samples have POLE with an inactivated exonuclease domain, leading to a loss of proofreading ability on newly-synthesized DNA (38,39). Samples with greater absolute numbers of mutations therefore will generally have either a stronger mutator phenotype, or have become *POLE* exonuclease domain mutated earlier. However, with neither of these factors expected to alter the rate of 5mC deamination, we hypothesized that exonuclease domain-mutated POLE may instead more often make replication errors when encountering a site requiring the insertion of guanine in a mCpG context, though other mechanisms of mutagenesis related to defective POLE may also be possible (40). (We note that these data could also be explained if errors are introduced by wild-type POLE when encountering a mCpG context, but we find no evidence in the literature from *in vitro* studies to suggest that wild-type replicative polymerases typically make such errors in the context of 5mCs).

With the TCG trinucleotide being the most highly mutated CpG variant in *POLE*-mutant tumours (3,41), we found a significantly greater proportion of T[C>T]G mutations to occur at high rather than low methylated TCG sites (*P* < 0.01, paired t-test; Figure 3B). Given POLE's role in leading strand replication (42,43), we investigated the strand-specificity of the T[C>A]T and T[C>T]G mutations, both of which are common mutations in *POLE*-mutant cancer genomes (3,41). We found significant strand asymmetry to occur in *both* trinucleotide contexts around two known origins of replication (*P* < 0.0001 and *P* < 0.05, Fisher's exact test; Figure 3C and D). This finding more likely associates CpG mutations in *POLE*-mutant tumors with mutagenesis, rather than with some unknown and genome-wide repair deficiency. As these mutations occur more often at methylated than unmethylated cytosines (Figure 1B and Figure 3B), we propose this mutagenesis to be specifically associated with 5mCs. Lending further support to our hypothesis is prior evidence suggesting that *POLE* exonuclease domain mutation can result in a mutator phenotype greater than that from proofreading-deficiency alone (39), with some variants increasing mutation load even above that from catalytic domain inactivation (41).

### mCpG mutations as potential driver events in *POLE*-mutant colorectal cancers

**Figure 4.**
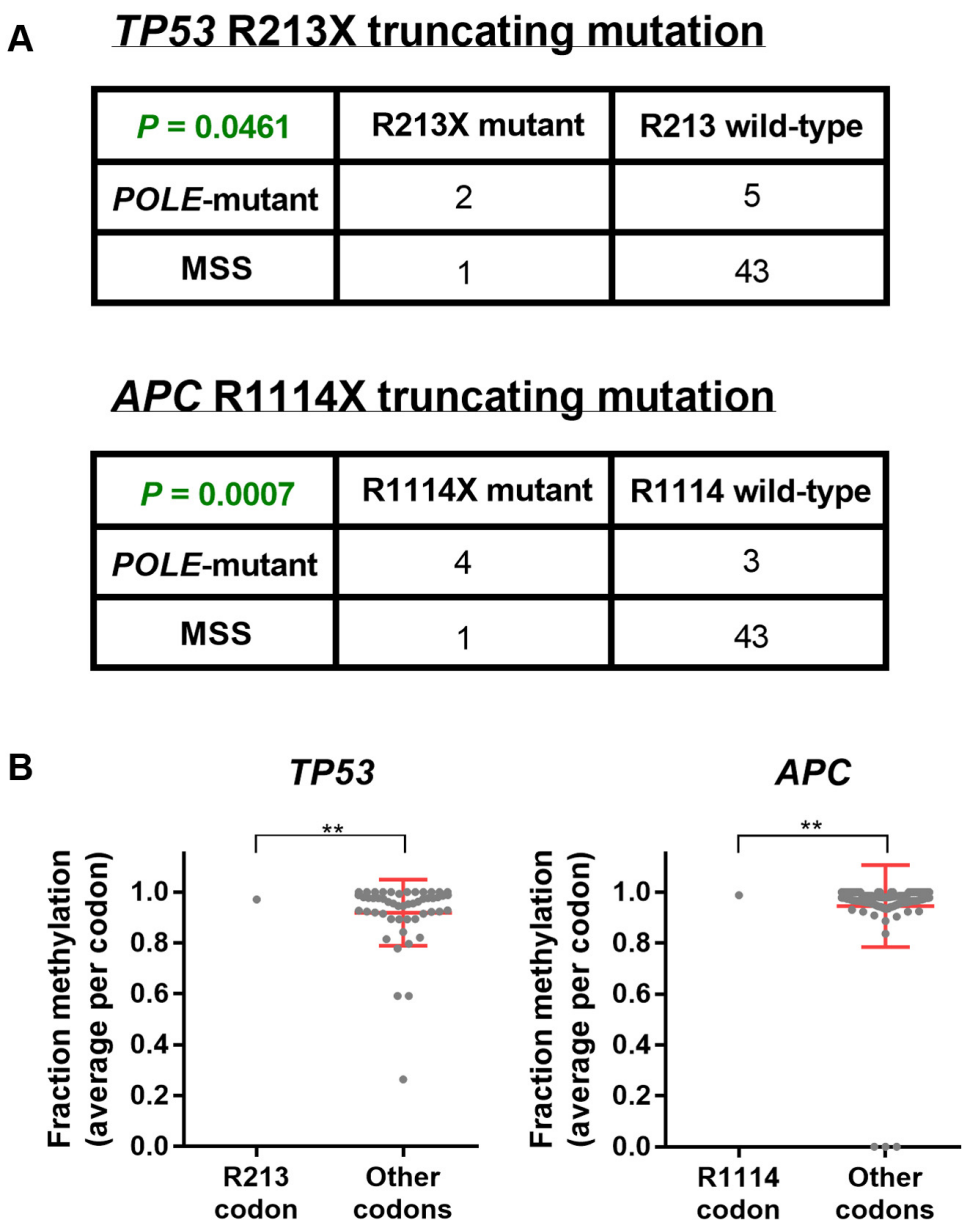
Mutation hotspots in cancer driver genes in *POLE*-mutant tumors. (**A**) Contingency table and significance from Fisher's exact test of *Polymerase epsilon* exonuclease domain mutant (*POLE*-mutant) and microsatellite stable (MSS) colorectal cancer samples which are wild-type or mutant at *tumor protein p53 (TP53)* R213 and *adenomatous polyposis coli (APC)* R1114 codons. (**B**) Methylation status in normal colon tissue for each CpG site within coding exons of *TP53* and *APC*, together with significance by one-sample t-test against methylation at R213 and R1114 codons respectively. Mean and standard deviation are shown. ** denotes *P* < 0.01.

Many mutations responsible for genetic diseases are C>T transitions occurring at CpG dinucleotides (15,44). Additionally, methylated CpGs are hotspots for somatic cancer mutations in driver genes such as *TP53, RB1* and *EGFR* (15,45–47). *POLE*-mutant colorectal cancers harbour specific mutation hotspots in the key tumour-suppressors *tumor protein p53 (TP53)* and *adenomatous polyposis coli (APC)* (41,48) (a finding which we have confirmed in our samples; Figure 4A). As *POLE* exonuclease domain mutation is thought to be an early event in tumors (38), these *POLE*-mutant-signature mutations could also occur early in oncogenesis, and serve as gatekeeper mutations—conferring a growth advantage to cellular subpopulations and driving tumor growth. We observed these mutation hotspots (truncating C>T mutations at *TP53* R213X and *APC* R1114X) to occur at TCG trinucleotides, leading us to hypothesize that these sites may be more often mutated specifically in *POLE*-mutant tumours because of the strong mutation–methylation association that we have observed in this cancer subtype.

We found these sites to be highly methylated in normal colon tissue, with the CpG at *TP53* R213 methylated in 97.1% of reads, and at *APC* R1114 methylated in 98.7% of reads (Figure 4B). However, while these sites are methylated to a significantly greater extent than other codons in the same gene (*P* < 0.01, one-sample *t*-test; Figure 4B), there may yet be other locations in *TP53* or *APC* which are equally likely to become mutated when considering methylation alone. To investigate this, we considered all possible C>T mutations at TCG trinucleotides which would lead to the immediate truncation of either *TP53* or *APC*. We found that the R213 site in *TP53* is the only possible trinucleotide fulfilling these criteria (Supplementary Figure S4A) and potentially explaining its hotspot mutation status in *POLE*-mutant samples. In *APC*, we found three additional sites occurring earlier from the N-terminal of the protein which fulfilled the criteria listed, together with five mutation sites at or after the C-terminal of codon 1920 (Supplementary Figure S4B). Taken together, our findings suggest that methylation may be responsible for the formation of specific mutation hotspots in *POLE*-mutant cancers, with other factors likely also contributing to mutation occurrence and selection within cells—perhaps due to a phenotype conferred to cells by mutations at specific sites which makes them more likely to be observed in cancer sequencing data (15).

### Differential influence of methylation on mutation accumulation across cancer types and subtypes

Having described a strong mutation–methylation association across colorectal cancer subtypes, we sought to investigate whether any such association exists in other cancer types. To do so, we incorporated into our analyses, somatic mutations from an additional 855 whole-genomes across 11 cancer types available from TCGA, ICGC and previously published datasets (3,22) (Supplementary Table S1A and Supplementary Table S1B). We developed regression models using both tissue type-specific methylation data (Supplementary Table S2) and average cell-type replication timing data, plotting actual mutations together with the function predicted by multivariable regression models (see Materials and Methods).

**Figure 5.**
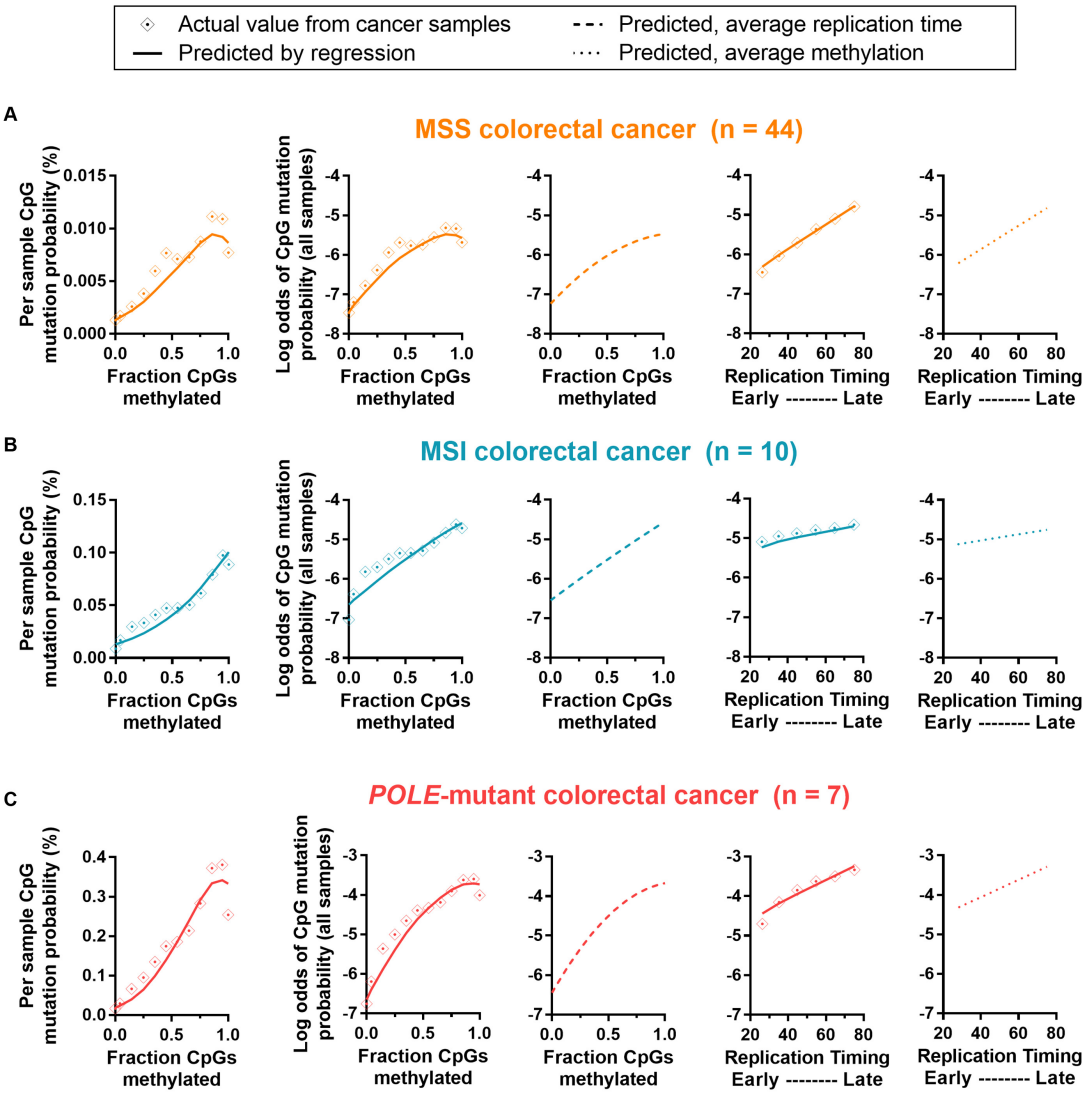
Actual and predicted mutation rates, according to methylation and replication timing for colorectal cancer subtypes. Graphs depict actual and predicted (by regression model; see Materials and Methods) mutation probability and log odds of mutation probability by methylation or replication timing, for (**A**) microsatellite stable (MSS) colorectal cancer, (**B**) colorectal cancers with microsatellite instability (MSI) and (**C**) colorectal cancers with *Polymerase epsilon* exonuclease domain mutation (*POLE*-mutant). Graphs from left to right are: mutation probability by fraction of CpGs methylated (actual and predicted), log odds of mutation probability by fraction of CpGs methylated (actual and predicted), log odds of mutation probability by fraction of CpGs methylated (predicted, using overall average replication timing in all bins), log odds of mutation probability by replication timing (actual and predicted) and log odds of mutation probability by replication timing (predicted, using overall average methylation in all bins). Binned data is shown (bins of 0.1 for methylation or 10 for replication timing). See Table 1 for regression output, predicted vertex and area under curve values.

To first validate our regression models, we investigated the predicted associations in colorectal cancer, finding a positive association between mutation probability and fraction of CpGs methylated across colorectal cancer subtypes for all possible methylation values (function vertex > 1 fraction CpGs methylated; Table 1), consistent with what we have already demonstrated. Also confirming previous findings (7), we found mutation probability to vary little across replication timing changes in MSI colorectal cancers, compared with MSS and *POLE*-mutant subtypes (depicted in rightmost graphs; Figure 5). This is also evident via the small improvement to the area under the curve (AUC) in nested models which additionally incorporated replication timing (MSI: 2.0%), compared with 16.4% in MSS and 12.6% in *POLE*-mutant subtypes (Table 1).

**Figure 6.**
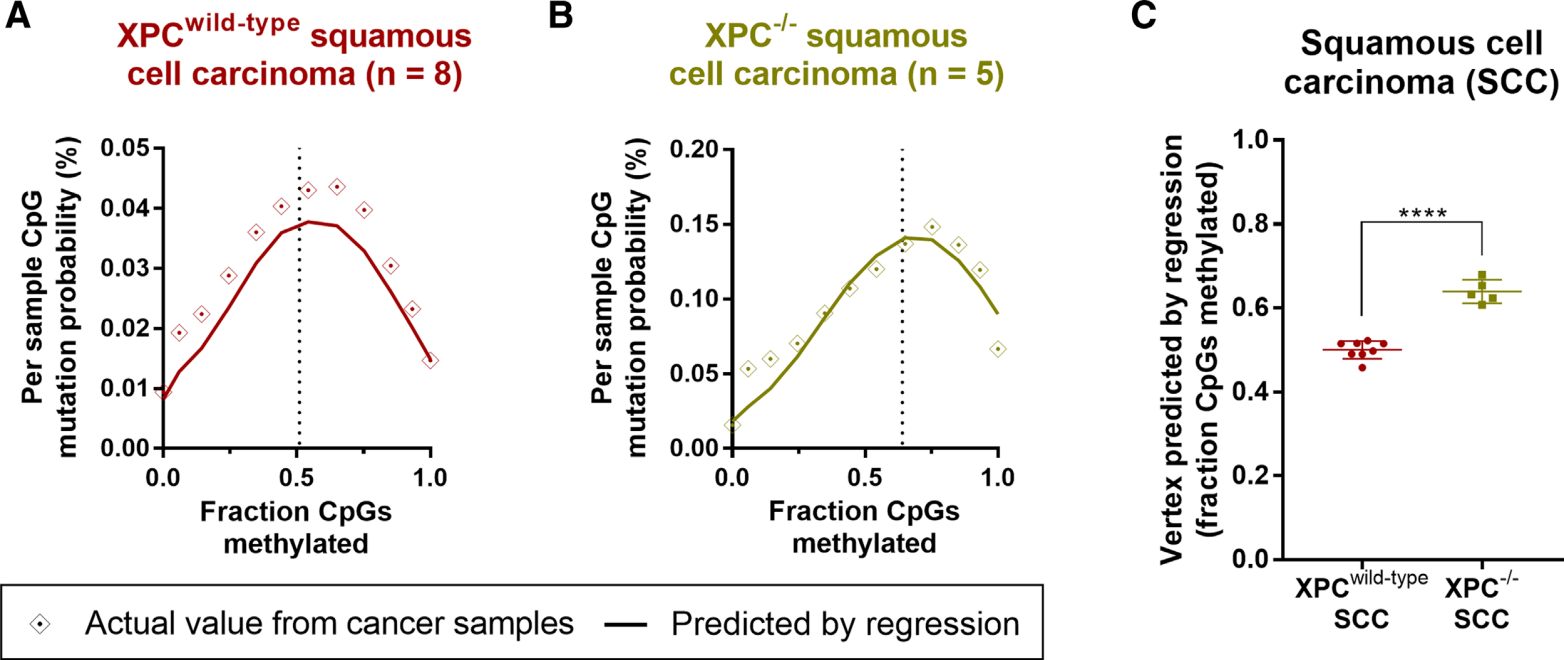
Actual and predicted mutation probability and regression function vertex according to methylation and replication timing for squamous cell carcinoma (SCC) subtypes. Graph depicting actual and predicted (by regression model; see Materials and Methods) mutation probability by fraction of CpGs methylated (actual and predicted) for (**A**) wild-type *Xeroderma pigmentosum complementation group C* (XPC*^wild-type^*) and (**B**) XPC mutant (XPC^−/−^) SCC. Binned data is shown (bins of 0.1 for methylation), with the function's vertex indicated by a dotted line. See Table 1 for regression output, predicted vertex and area under curve values. (**C**) Vertex predicted by regression model on individual XPC*^wild-type^* and XPC^−/−^ SCC, with significance by unpaired *t*-test. Mean and standard deviation are shown. **** denotes *P* < 0.0001.

Having validated our regression models in this way, we then examined the mutation–methylation association in skin cancer subtypes, as skin cancers are subject to well-defined mutation and repair processes associated with UV light. The propensity for mutagenic cyclobutane pyrimidine dimer (CPD) DNA lesion formation following UV light exposure is known to increase at mCpGs (49,50), and hence we would expect that the underlying association between CpG mutation rate and methylation in UV light-induced cancers should be both positive and linear. However, in both squamous cell carcinoma (SCC) and melanoma we found that the association between mutation rate and methylation was non-linear (Figure 6A and Supplementary Figure S5G). The vertex predicted by our multivariable regression model was at 0.51 (SCC; Table 1) and 0.50 (melanoma; Supplementary Table S3) fraction of CpGs methylated, meaning that at methylation fractions greater than ∼0.5, increasing methylation was actually associated with decreasing mutation probability (Figure 6A and Supplementary Figure S5G).

To determine why this might be the case, we examined the efficiency of NER in the context of replication timing. NER is a key repair mechanism in many skin cancers due to its role in the removal of UV light-induced DNA lesions We find levels of NER (in response to UV light exposure) to be increased in early-replicating regions (CPD: *r*^2^ = 0.78, and (6–4)pyrimidine–pyrimidone photoproduct ((6–4)PP): *r*^2^ = 0.57, *P* < 0.0001, Pearson's correlation; Supplementary Figure S6A), likely due to early-replicating regions tending to be more highly-transcribed (51) and therefore more frequently subject to transcription-coupled NER and domain-associated global genome NER (22). We found that the mutation–methylation pattern in the skin cancers (Figure 6A and Supplementary Figure S5G) closely mimics the replication timing-methylation pattern in NHEK cells (Supplementary Figure S6B), suggesting that NER may underlie the non-linear relationship between methylation and CpG mutation rate in skin cancers. To investigate this, we examined the mutation–methylation association in global genome NER-deficient *Xeroderma pigmentosus complementation group C* mutant (XPC^−/−^) SCCs (Figure 6B and Supplementary Figure S6D), and compared this with XPC^wild-type^ SCCs (Figure 6A and Supplementary Figure S6C). Although the mutation–methylation association also remained non-linear in the NER-deficient XPC^−/−^ cancer sub-type, we found the vertex of the function predicting mutation probability to have shifted upwards from 0.51 in XPC^wild-type^ SCC, to 0.64 fraction of CpGs methylated in XPC^−/−^ SCC (Table 1). [This shift can be reproduced with vertices predicted by regression models using individual XPC^wild-type^ and XPC^−/−^ SCC samples (*P* < 0.0001, unpaired *t*-test; Figure 6C)]. Further, the AUC showed a 4.9% improvement when methylation was added to a nested model in XPC^−/−^ SCC, with only a 2.4% improvement in XPC^wild-type^ SCC (Table 1). Taken together, our results suggest that the negative association between mutation rate and methylation at high fractions of methylation is, at least in part, driven by the underlying mutation-replication timing-association induced by NER reliance following UV light exposure. As some highly-methylated regions are active gene bodies which tend to be both early-replicating (52) and subject to transcription-coupled NER (22), this likely leads to their reduced overall mutation load in skin cancers.

When investigating other cancer types, the multivariable regression models predicted the regression function's vertex to be between 0 and 1 fraction of CpGs methylated for breast, liver, ovarian and pancreatic cancers, as well as chronic lymphocytic leukaemia (Supplementary Table S3, Supplementary Figure S5). The primary mutation and repair processes are not well understood in many of these cancers, with samples harbouring varied mutation signatures and many mutations of unknown origin (3). It is possible that our regression models are unable to completely separate the association between replication timing and methylation (with both factors significantly impacting on mutation rate), or that tumour-specific methylation changes significantly alter the mutation–methylation associations that we observe. However, it may also be true that in some cancer types, the underlying association with methylation is actually such that, at high rates of methylation, mCpGs are in fact less likely to become mutated, due to the specific mutation and repair processes inherent in various tissue types. In fact, other analyses have shown that the genome-wide rate of C>T single nucleotide polymorphisms (SNPs) increases only at low and intermediate (20–60%) methylated sites, but not at sites with high methylation (53).

## CONCLUSION

In this study, we analysed 61 colorectal cancer whole-genomes, together with data from an additional 11 cancer types. Using tissue-specific methylation data, we describe a strong association between C>T mutations and methylation at CpG dinucleotides in many cancer types, driving patterns of mutation formation throughout the genome. Our evidence suggests that MMR may play a role in the correction of G•T mismatches resulting from deamination of 5mC. We also propose a mutator phenotype occurring specifically at 5mCs that results from *POLE* exonuclease domain mutation—a phenotype that we implicate in potentially driving tumour growth through the formation of specific mutation hotspots in key cancer-associated genes. Additionally, we reveal distinct associations between mutation and methylation across cancer types, highlighting the influence of DNA repair on mutation–methylation associations in some genomes. Together, our findings provide significant developments to our understanding of mutation formation and repair at CpG dinucleotides in cells.

Our study describes distinct mutation–methylation associations in cancer genomes which must be understood in order to effectively predict expected mutation loads across cancer types and subtypes. We emphasize the need for researchers to understand and stratify cancer subtypes according to relevant mutation and repair mechanisms when developing predictive models of mutation rates according to genetic and epigenetic features in the genome. Doing so will allow scientists to more accurately distinguish driver from passenger mutations. Our findings reveal novel links between methylation and common mutation and repair processes, as we show these to be key mechanisms that define the mutational landscape of cancer genomes.

## Supplementary Material

Supplementary Data
