# Supplementary material for: The interaction between cytosine methylation and processes of DNA replication and repair shape the mutational landscape of cancer genomes
Source: Nucleic Acids Res. 2017 May 22;45(13):7786–95. doi: 10.1093/nar/gkx463 (PMC5737810; doi:10.1093/nar/gkx463)
Supplement: Supplementary Data [file gkx463_supp.zip › Supplementary_data_FINAL.pdf]

Supplementary Material

Supplementary Materials and Methods

See *Materials and Methods* in the main manuscript for further information.

Supplementary Figures

- **Supplementary Figure S1** – Mutation, DNase I hypersensitivity (DHS) and H3K4me3 signal around transcription start sites (TSSs) in colon cells.
- **Supplementary Figure S2** – Association between mutation accumulation and methylation across changes in replication timing in colorectal cancers with *polymerase epsilon (POLE)* exonuclease domain mutations
- **Supplementary Figure S3** – Association between mutations and methylation in individual *POLE*-mutant colorectal cancers.
- **Supplementary Figure S4** – Methylation status for all possible sites of truncating T[C>T]G mutations within *tumor protein p53 (TP53)* and *adenomatous polyposis coli (APC)* genes in normal colon tissue.
- **Supplementary Figure S5** – Actual and predicted mutation rates, according to methylation and replication timing, across cancer types.
- **Supplementary Figure S6** – Nucleotide excision repair, methylation and regression models in skin cells.

Supplementary Tables

- **Supplementary Table S1A** – Cancer types and data sources for all somatic mutations analysed. (See excel file for **Supplementary Table S1B**).
- **Supplementary Table S2** – Methylation datasets matched with each cancer type and subtype used in regression analyses.
- **Supplementary Table S3** – Regression equation from multivariable models predicting mutation probability across cancer types and subtypes, together with vertex and area under curve (AUC) predictions.

Supplementary Data

- **Supplementary Data Note**
- **Supplementary Data Table** – Deletion mutations in *MBD4* coding exons in MSI colorectal cancer samples, together with segment mean (indicating copy number) at the locus.
- **Supplementary Data Figure** – Evaluation of base excision repair (BER) and mCpG mutation rate in colorectal cancers with microsatellite instability (MSI).

Supplementary Material References

Reference list for all citations included in Supplementary Material.

Supplementary Materials and Methods

Classification of cancer subtype sand signature mutations

Samples with microsatellite instability (MSI) were selected if they were listed as "MSI-H" in TCGA "clinical" data from the TCGA data portal. *Polymerase epsilon (POLE)* exonuclease domain mutated (*POLE*-mutant) colorectal cancer samples were selected as such if they had a genome-wide mutation signature correlation with Signature 10 (1) of greater than 0.85. Sample classifications were confirmed as exonuclease domain mutated if they contained an exonuclease domain mutation (between codons 268-471) (2) as listed by Shinbrot et al. (2014) in "Supplemental Table 1A" (3).

Squamous cell carcinoma (SCC) samples were determined to be *Xeroderma pigmentosus complementation group C* (XPC) wild-type or mutant ($XPC^{-/-}$) based on classifications made by Zheng et al (2014) (4).

Gene profiles and data binning

H3K4me3 chromatin immunoprecipitation sequencing (ChIP-seq) data from normal sigmoid colon tissue were obtained from the Roadmap Epigenomics Atlas (5) (GEO: GSM956024), and converted to bigwig using "wigToBigWig". Normal sigmoid colon tissue whole genome bisulfite sequencing (WGBS) methylation data were as previously described (see *Materials and Methods*), and converted to bigwig similarly. DNase I hypersensitivity (DHS) data were obtained for HCT116 cells from the ENCODE project consortium (6) and downloaded as a bigwig file through the UCSC Genome Browser (GEO: GSM736600).

Transcription start sites (TSSs) for each gene were obtained from the UCSC Table Browser. Mutation profiles were generated by counting mutations at each base within ± 4 kb of a TSS, with counts normalised to mutations per Mb. Methylation, H3K4me3 and DHS

profile data were created by use of the “computeMatrix” (reference-point) and “plotProfile” tools available through the deepTools package (7). All profiles were orientated so that the gene body runs from 5’ to 3’, downstream from the TSS.

Where data are binned across methylation values, bins spanning 0.1 methylation were used for all methylation values between 0 and 1. For CpGs with methylation values equalling exactly 0 or 1, data were allocated to bins representing either 0 or 1 methylation only, respectively. Regression models and correlations which incorporated methylation data (that is, **Fig 1b**, **2**, **5**, **6a-b** and **Supp Fig S2**, S**3**, S**5**, **S6c-d**) used only methylation values from CpG dinucleotides located on autosomes.

To calculate mutation-methylation associations in regions of the genome with differing replication timing (**Fig 2** and **Supp Fig S2**), regions were determined to be early-replicating (70-80 replication timing), mid-replicating (45-55 replication timing), and late-replicating (20-30 replication timing). Sites were excluded from analyses in **Fig 2**, **Supp Fig S2** and **Supp Fig S6a-b** if they had replication timing listed as < 20 or > 80, to ensure robust measurements with enough data points. For presentation of replication timing data in regression model figures (**Fig 5**, **6a-b** and **Supp Fig S5**, **S6c-d**), replicating timing values have been inverted, such that lower values indicate earlier replication. chrY was excluded from replication timing analyses, as values for this chromosome were not present in the original raw data.

Strand specificity and origins of replication

TOP1 and *LMNB2* origin of replication (oriC) sites were selected for use since they were well-defined oriC per Shinbrot et al. (2014) (3). The region used in analyses as that surrounding the oriC for *TOP1* was chr20:39,300,000-39,900,000, with the oriC isolated upstream of the *TOP1* TSS as shown in **Fig 3d** (upper panel). The region used in analyses as

that surrounding the oriC for *LMNB2* was chr19:2,000,000-2,700,000, with the oriC as given in Shinbrot et al. (2014) (3) and shown in **Fig 3d** (lower panel).

Further details of regression models and other statistical analyses

To plot the actual and predicted values from the regression model (as in **Fig 5, Fig 6a-b, Supp Fig S5** and **Supp Fig S6c-d**), data was binned either by methylation (bin size of 0.1, ranging from 0 to 1; for CpGs with methylation values equalling exactly 0 or 1, mutations were allocated to bins representing 0 or 1 methylation only, respectively) or by replication timing (bin size of 10, ranging from 20 to 80). Where mutation probability or log odds of mutation probability was plotted against methylation, an average was used for replication timing within each bin. To separate the influence of each factor, where the predicted function was plotted against methylation using average replication timing or against replication timing using average methylation, the overall genome-wide average for replication timing or methylation (respectively) was used in the equation for all bins. Where log odds of mutation probability was plotted against replication timing, an average was used for methylation within each bin.

In **Fig 1b,** significance was determined by Pearson's correlation on binned data, with slopes of MSI and *POLE*-mutant colorectal cancer compared to MSS colorectal cancer data via a linear regression, with MSS as the factor level reference. In **Fig 2** and **Supp Fig S2**, the same analysis was performed, with slopes of mid- and late-replicating regions compared to early-replicating regions, with early-replication as the factor level reference. Statistical analyses displayed in **Fig 3a** and **Supp Fig S6a** were performed using Pearson's correlation on the data points shown. Fisher's exact test was used to determine the levels of significance given in **Fig 3c** and **Fig 4a**. **Fig 3b** shows significance by paired t-test between samples and **Fig 4b** displays significance by one-sample t-test. All other determinations of significance

were made by unpaired t-test. In all instances, significance was determined using a threshold of $P < 0.05$.

Methods relating to Supplementary Data Note

Somatic indels were identified using SvABA (Structural Variation Analysis By Assembly; https://github.com/walaj/svaba) with default parameters. The tool was run on the Seven Bridges Cancer Genomics Cloud (CGC) (cgc.sbgenomics.com). Copy number segment information for MSI colorectal cancer samples were obtained from the Genomic Data Commons (GDC). Samples were deemed to have a copy number of two if the segment mean was between -0.2 and 0.2.

Supplementary Figure S1

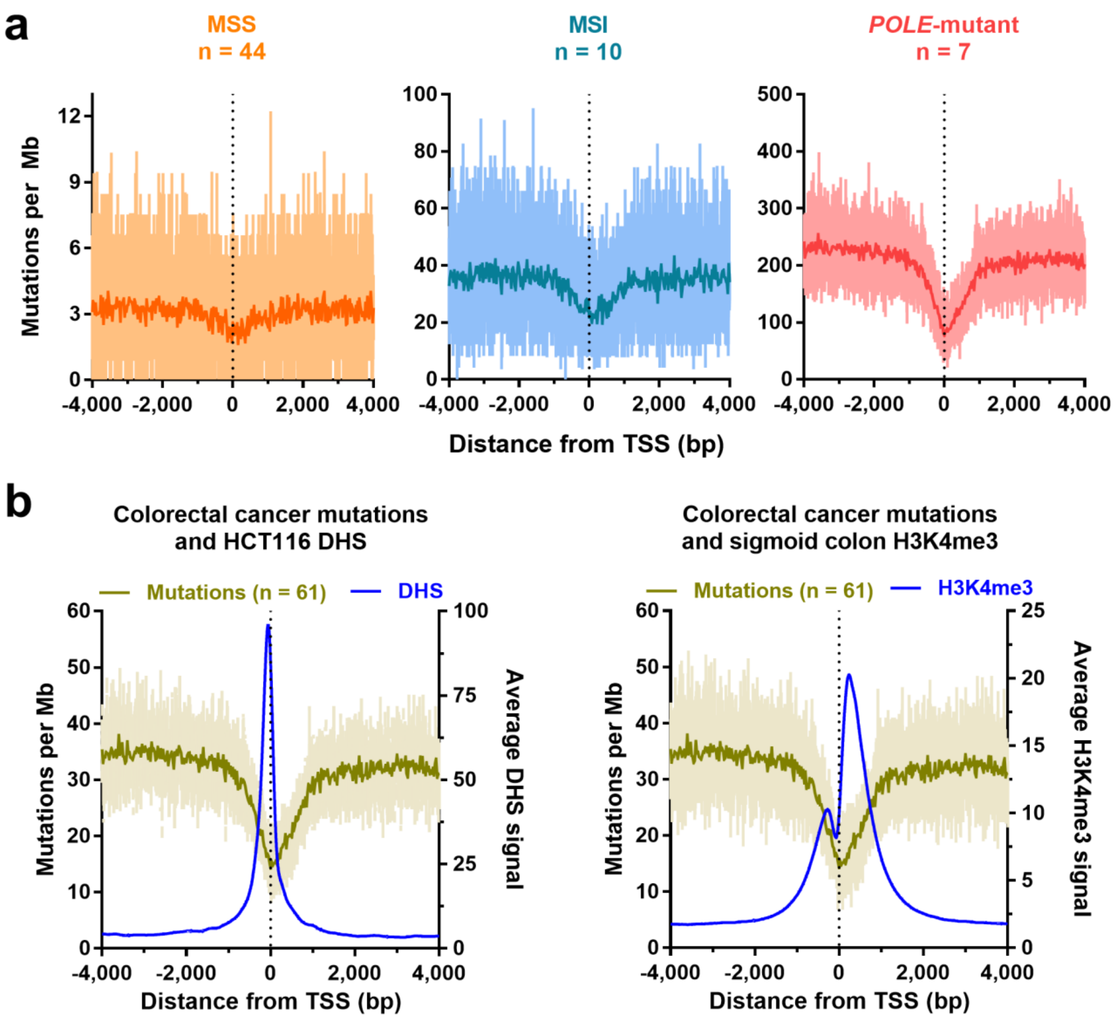

Supplementary Figure S1 – Mutation, DNase I hypersensitivity (DHS) and H3K4me3 signal around transcription start sites (TSSs) in colon cells. (a) Mutation profiles around the TSS for microsatellite stable (MSS) colorectal cancer (left panel), those with microsatellite instability (MSI; middle panel) and with *Polymerase epsilon* exonuclease domain mutation (*POLE*-mutant; right panel). For mutations, nucleotide-resolution data is shown (light colour) along with data in 25 bp bins (dark colour). **(b)** Colorectal cancer mutation profile along with average DHS signal from HCT116 colorectal cancer cell-line (left panel) or colon tissue H3K4me3 signal (right panel) around the TSS.

Supplementary Figure S2

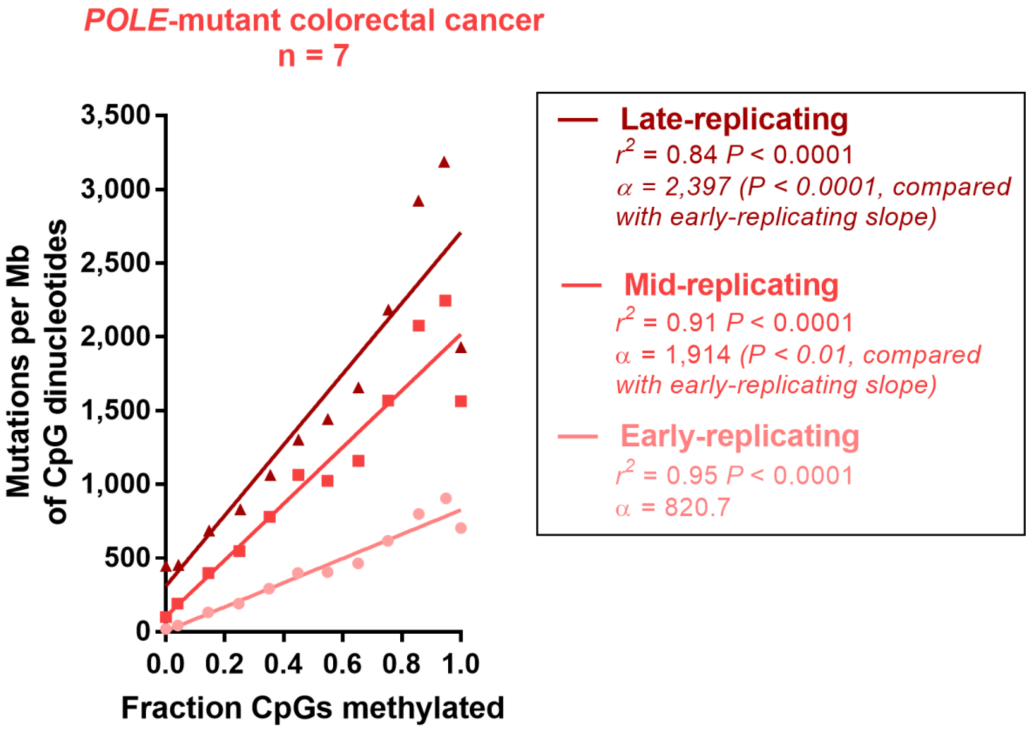

Supplementary Figure S2 - Association between mutation accumulation and methylation across changes in replication timing in colorectal cancers with *polymerase epsilon (POLE)* exonuclease domain mutations. Correlation between mutations per megabase (Mb) at CpG dinucleotides and fractions of CpGs methylated (using normal sigmoid colon tissue WGBS methylation data) across autosomes in *POLE*-mutant colorectal cancers. Genome-wide data is binned for each colorectal cancer subtype (bins of 0.1 methylation), along with r^2 and significance from Pearson's regression. The comparison of mid- and late-replicating slopes with early-replicating slopes was calculated by linear regression on binned data, with "early-replicating" as the reference factor.

Supplementary Figure S3

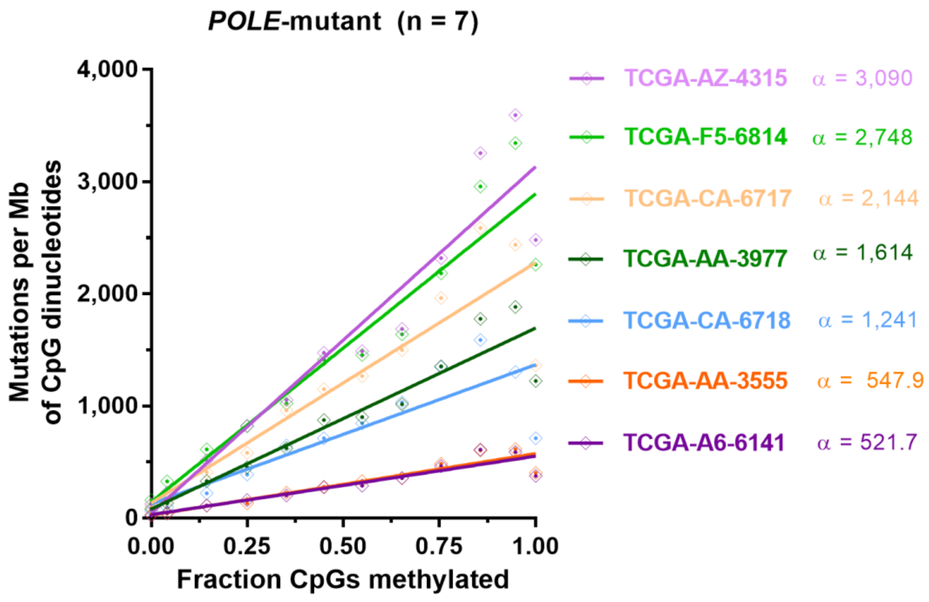

Supplementary Figure S3 – Association between mutations and methylation in individual *POLE*-mutant colorectal cancers. Line of best fit from binned data of mutation-methylation associations in *Polymerase epsilon* exonuclease domain mutant (*POLE*-mutant) colorectal cancers. α denotes the slope of the line of best fit, with data binned by 0.1 methylation.

Supplementary Figure S4

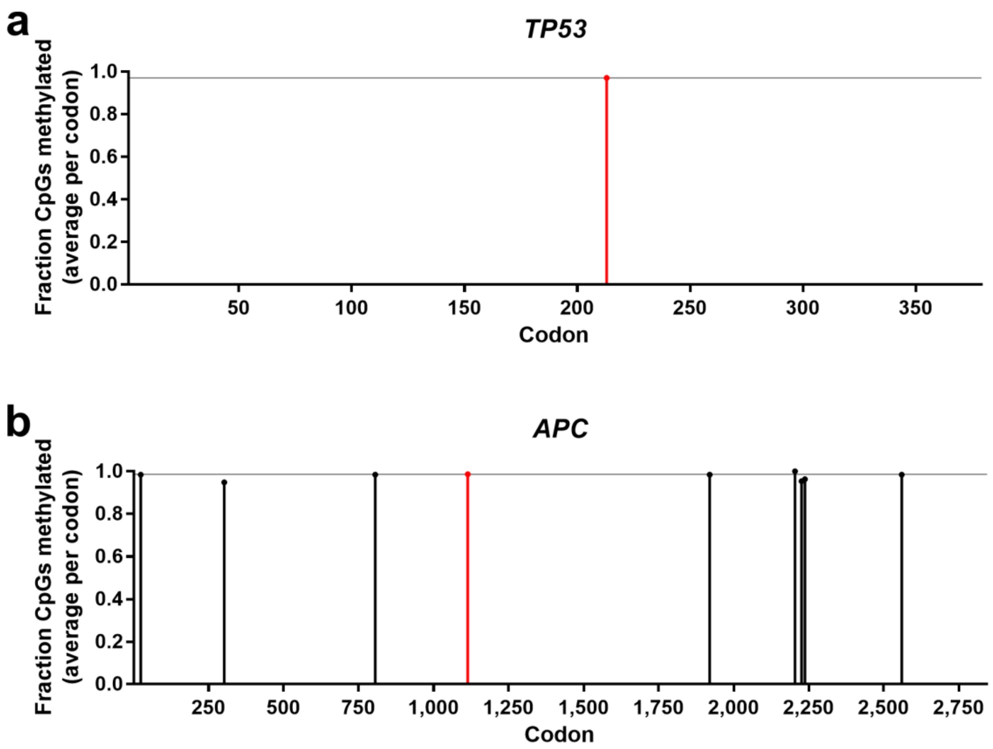

Supplementary Figure S4 – Methylation status for all possible sites of truncating T[C>T]G mutations within *tumor protein p53 (TP53)* and *adenomatous polyposis coli (APC)* genes in normal colon tissue. Methylation status in normal colon tissue of each TCG trinucleotide which, via a C>T mutation, would result in a protein truncation within a coding exon of **(a)** *TP53* and **(b)** *APC*. The R213 (*TP53*) and R1114 (*APC*) codons are indicated in red, with a horizontal line marking the fraction CpGs methylated level at these codons.

Supplementary Figure S5

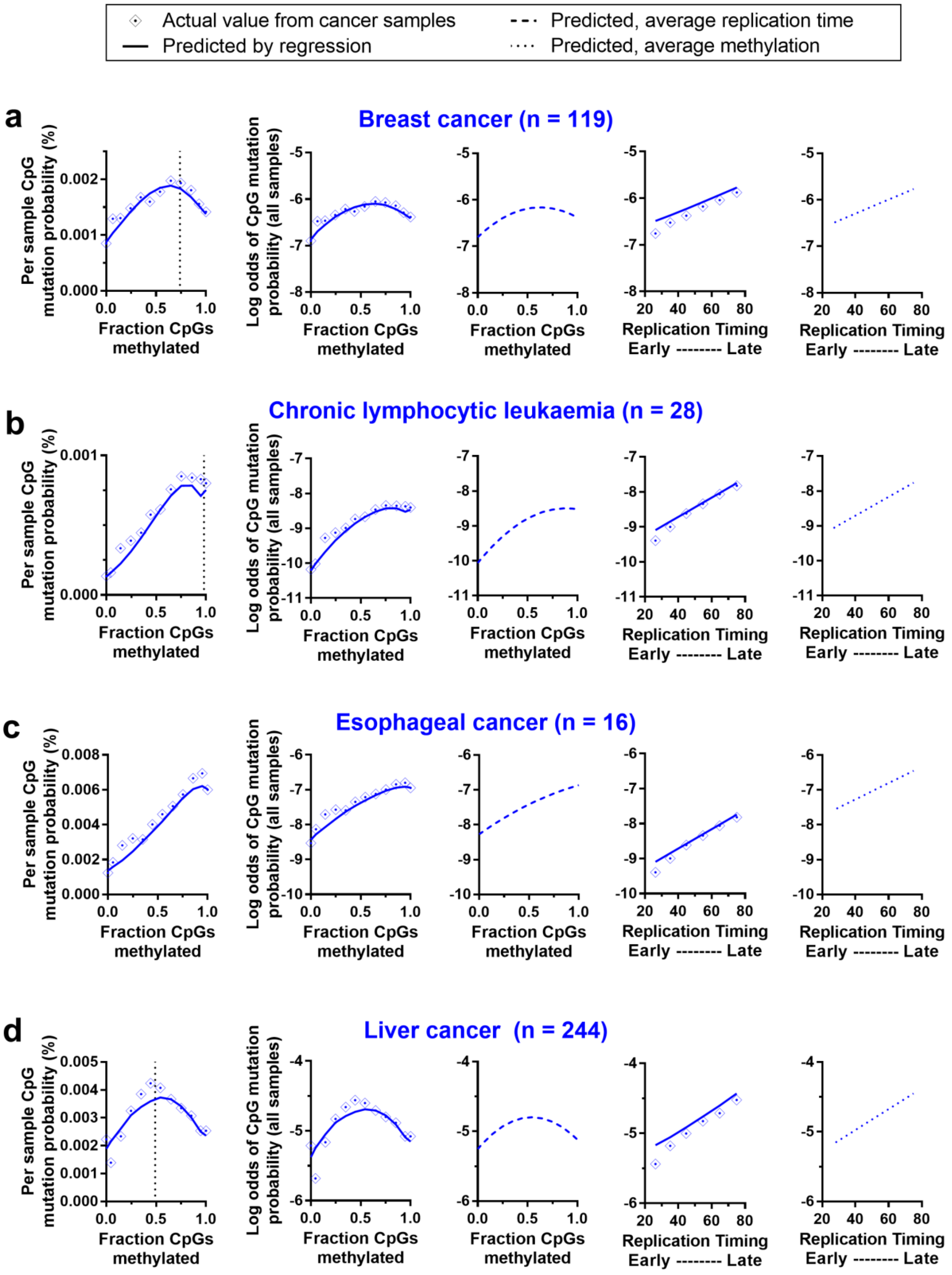

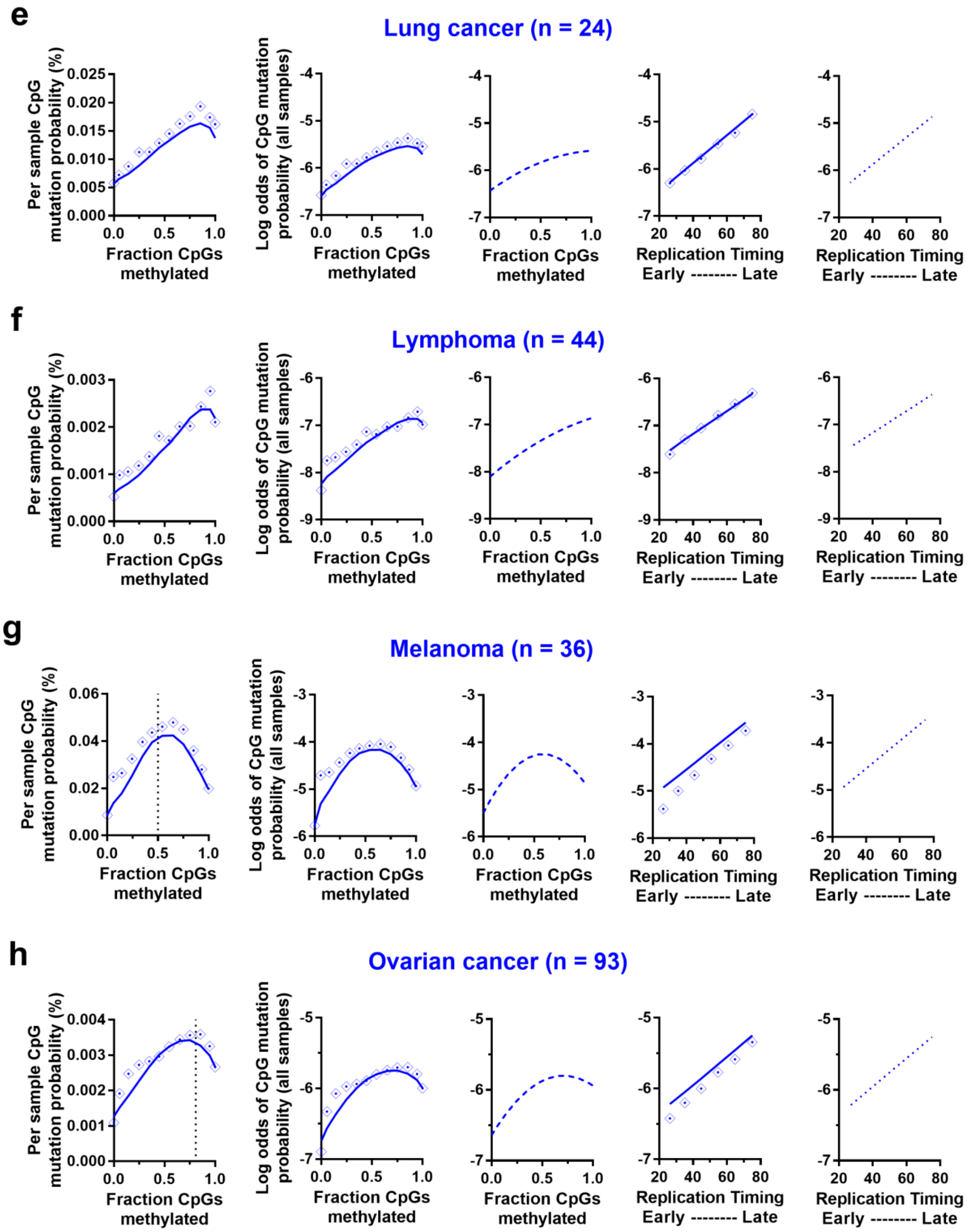
e
Lung cancer (n = 24)
f
Lymphoma (n = 44)
g
Melanoma (n = 36)
h
Ovarian cancer (n = 93)
Per sample CpG mutation probability (%)
Log odds of CpG mutation probability (all samples)
Fraction CpGs methylated
Replication Timing
Early -------- Late

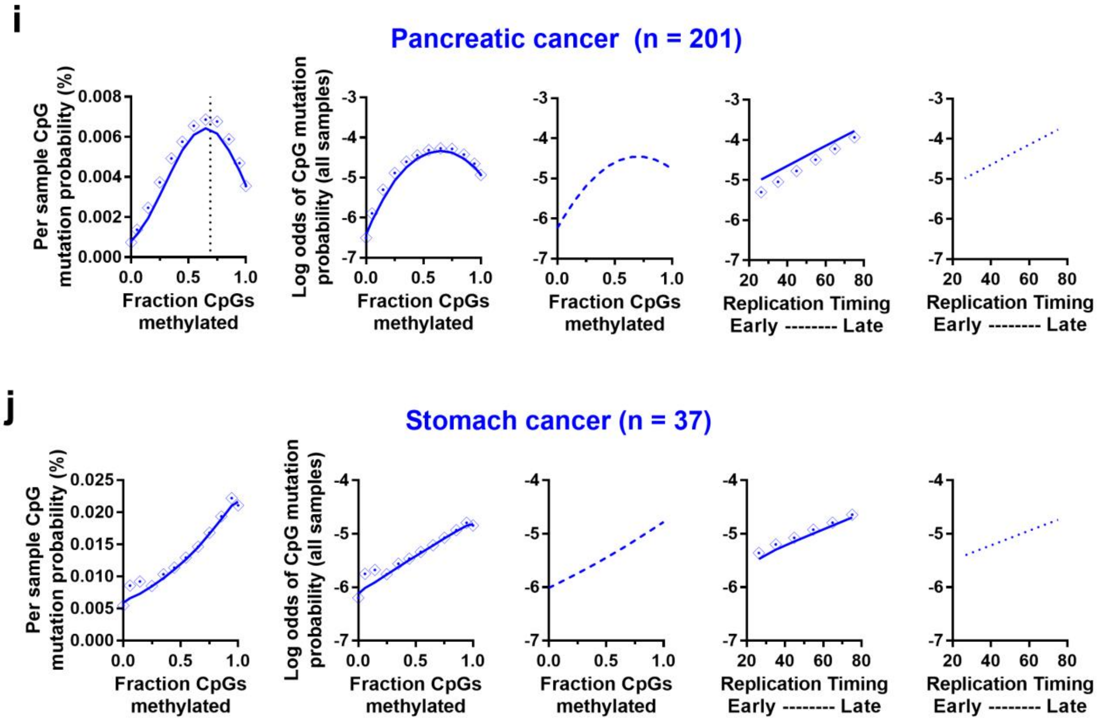

Supplementary Figure S5 – Actual and predicted mutation rates, according to methylation and replication timing, across cancer types. Graphs depict actual and predicted (by regression model; see **Materials and Methods**) mutation probability and log odds of mutation probability by methylation or replication timing, for **(a)** breast cancer, **(b)** chronic lymphocytic leukaemia, **(c)** esophageal cancer, **(d)** liver cancer, **(e)** lung cancer, **(f)** lymphoma, **(g)** melanoma, **(h)** ovarian cancer, **(i)** pancreatic cancer and **(j)** stomach cancer. Graphs from left to right are: mutation probability by fraction CpGs methylated (actual and predicted), log odds of mutation probability by fraction CpGs methylated (actual and predicted), log odds of mutation probability by fraction CpGs methylated (predicted, using overall average replication timing in all bins), log odds of mutation probability by replication timing (actual and predicted) and log odds of mutation probability by replication timing (predicted, using overall average methylation in all bins). Binned data is shown (bins of 0.1 for methylation or 10 for replication timing), with any vertex between 0 and 1 fraction CpGs methylated indicated by a dotted line. See **Supp Table S3** for regression output, predicted vertex and area under curve values.

Supplementary Figure S6

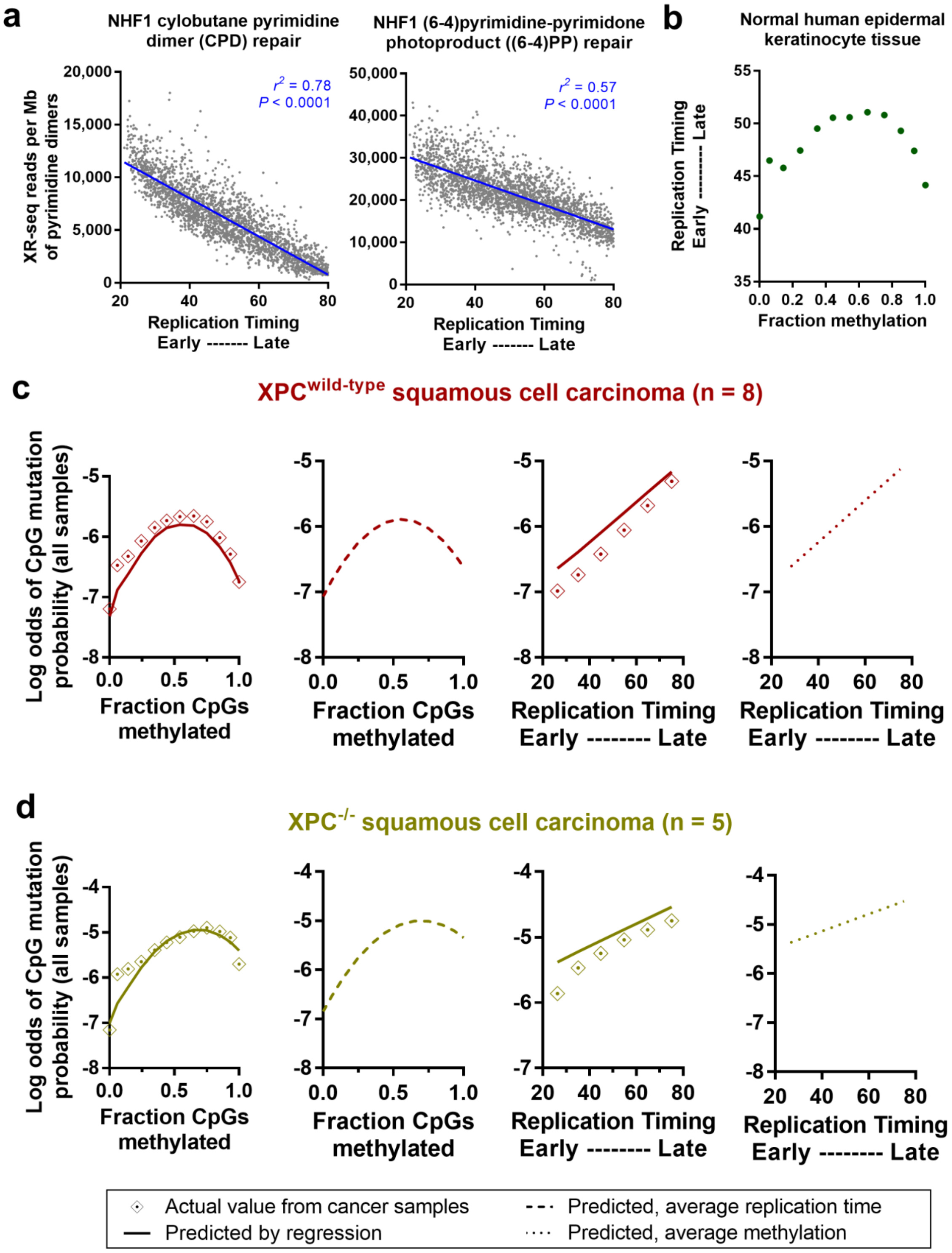

Supplementary Figure S6 – Nucleotide excision repair, methylation and regression models in skin cells. (a) Association between cyclobutane pyrimidine dimer (CPD; left) or

(6-4)pyrimidine-primidone photoproduct ((6-4)PP; right) excision sequencing (XR-seq) repair reads per megabase (Mb) of pyrimidine dimers with replication timing. r^2 and significance is by Pearson's correlation, with grey dots showing data binned to megabase scales. **(b)** Association between replication timing and normal human epidermal keratinocyte (NHEK) cell methylation, with data shown in bins of 0.1 methylation. Graphs depicting actual and predicted (by regression model; see **Materials and Methods**) log odds of mutation probability by methylation or replication timing for **(c)** $XPC^{wild\text{-}type}$ and **(d)** $XPC^{-/-}$ squamous cell carcinoma. Graphs from left to right are: log odds of mutation probability by fraction CpGs methylated (actual and predicted), log odds of mutation probability by fraction CpGs methylated (predicted, using overall average replication timing in all bins), log odds of mutation probability by replication timing (actual and predicted) and log odds of mutation probability by replication timing (predicted, using overall average methylation in all bins). Binned data is shown (bins of 0.1 for methylation or 10 for replication timing), with any vertex between 0 and 1 fraction CpGs methylated indicated by a dotted line. See **Table 1** for regression output, predicted vertex and area under curve values.

Supplementary Tables

Supplementary Table S1A – Cancer types and data sources for all somatic mutations analysed.

Cancer type	Cancer name used in manuscript	Source*
Breast invasive carcinoma	Breast cancer	Alexandrov et al. (1)
Chronic lymphocytic leukemia	Chronic lymphocytic leukaemia	Alexandrov et al. (1)
Colorectal adenocarcinoma	Colorectal cancer	TCGA
Esophageal adenocarcinoma	Esophageal cancer	ICGC
Liver hepatocellular carcinoma	Liver cancer	ICGC
Lung adenocarcinoma	Lung cancer	Alexandrov et al.(1)
Malignant non-Hodgkin lymphoma	Lymphoma	ICGC
Skin cutaneous melanoma	Melanoma	TCGA
Ovarian serous cystadenocarcinoma	Ovarian cancer	ICGC
Pancreatic ductal adenocarcinoma	Pancreatic cancer	ICGC
Squamous cell carcinoma	$XPC^{wild\text{-}type}$ squamous cell carcinoma (SCC)	Zheng et al. (4)
$XPC^{-/-}$ squamous cell carcinoma	$XPC^{-/-}$ squamous cell carcinoma (SCC)	Zheng et al. (4)
Stomach Adenocarcinoma	Stomach cancer	TCGA

* TCCA = The Cancer Genome Atlas; ICGC = International Cancer Genome Consortium

See excel file for **Supplementary Table S1B** which contains sample IDs for all cancers examined in this study.

Supplementary Table S2 – Methylation datasets matched with each cancer type and subtype used in regression analyses.

Cancer Type	Samples*	WGBS tissue type#	GEO+ accession
Breast cancer	119	Breast luminal epithelial cells	GSM1127125
Chronic lymphocytic leukaemia	28	CD34, mobilized primary cells	GSM916052
Esophageal cancer	16	Esophagus, adult	GSM983649
Liver cancer	244	Liver, adult	GSM916049
Lung cancer	24	Lung, adult	GSM983647
Lymphoma	44	Thymus	GSM1010979
Melanoma	36	Foreskin keratinocyte primary cells	GSM1127056
Ovarian cancer	93	Ovary, adult	GSM1010980
Pancreatic cancer	201	Pancreas	GSM983651
Squamous cell carcinoma	13	Foreskin keratinocyte primary cells	GSM1127056
Stomach cancer	37	Gastric, adult	GSM1010984

* See **Supp Table S1A** for data sources.
# WGBS = whole genome bisulfite sequencing. All tissue-types used were from normal cells.
+ GEO = Gene Expression Omnibus

Supplementary Table S3 – Regression equation from multivariable models predicting mutation probability across cancer types and subtypes, together with vertex and area under curve (AUC) predictions.

Cancer type/subtype	Regression model equation*	Vertex^	AUC@ for regression model A[A]	AUC@ for regression model B[B]	AUC@ for regression model C[C]	Increase to AUC via replication#	Increase to AUC via methylation+
Breast cancer	$y = -6.2782 + 2.3895M + -1.6076M^2 + -0.01*R + -0.0069(M \times R)$	0.74	0.550	0.584	0.594	8.0%	1.7%
Chronic lymphocytic leukaemia=	$y = -8.9527 + 3.9184M + -2.0027M^2 + -0.0212*R + -0.0072(M \times R)$	0.98	0.557	0.649	0.666	19.7%	2.7%
Esophageal cancer=	$y = -7.1719 + 2.1664M + -0.5986M^2 + -0.0211*R + -0.0031(M \times R)$	1.81	0.561	0.622	0.644	14.8%	3.5%
Liver cancer	$y = -4.3453 + 1.5195M + -1.5371M^2 + -0.0172*R + 0.0029(M \times R)$	0.49	0.555	0.585	0.593	6.8%	1.3%
Lung cancer	$y = -5.2744 + 1.9996M + -0.7005M^2 + -0.0221*R + -0.0087(M \times R)$	1.43	0.552	0.648	0.657	19.0%	1.4%
Lymphoma	$y = -7.1358 + 2.09M + -0.565M^2 + -0.0185*R + -0.0055(M \times R)$	1.85	0.564	0.624	0.639	13.4%	2.5%
Melanoma	$y = -3.5202 + 3.6233M + -3.5961M^2 + -0.0379*R + 0.0117(M \times R)$	0.50	0.595	0.659	0.674	13.4%	2.4%
Ovarian cancer	$y = -5.8518 + 2.7244M + -1.6735M^2 + -0.015*R + -0.0068(M \times R)$	0.81	0.552	0.608	0.620	12.3%	2.0%
Pancreatic cancer	$y = -4.8767 + 4.9924M + -3.6038M^2 + -0.026*R + 0.0015(M \times R)$	0.69	0.592	0.634	0.658	11.0%	3.7%
Stomach cancer	$y = -5.2204 + 0.9192M + 0.2171M^2 + -0.0151*R + 0.0018(M \times R)$	-2.12	0.573	0.571	0.606	5.8%	6.3%

* See **Materials and Methods** for canonical regression model formula, where y = log odds of mutation probability, M = methylation and R = replication timing

^ Vertex (unit: fraction CpGs methylated) predicted by regression model, calculated as $-b_1/(2\times b_2)$.

@AUC = area under curve

[A] Regression model equation: $y = b_0 + b_1 M + b_2 M^2$ where y = log odds of mutation probability, M = methylation and R = replication timing
[B] Regression model equation: $y = b_0 + b_1 R$ where y = log odds of mutation probability, M = methylation and R = replication timing
[C] Regression model equation: $y = b_0 + b_1 M + b_2 M^2 + b_3 R$, where y = log odds of mutation probability, M = methylation and R = replication timing
[#] Calculated using values of AUC from models: (C-A)/A
[+] Calculated using values of AUC from models: (C-B)/B
[=] For both chronic lymphocytic leukaemia (CLL) and esophageal cancers, the LRT did not indicate that there was a significant improvement between the model given above but excluding the interaction term $(M \times R)$, and the full model as stated above, including the interaction term ($P = 0.1303$ and $P = 0.1055$, respectively). However, as the AIC was still smallest for the full model which included the interaction term, and for consistency of model usage across cancer types, the full model was used for the analyses of CLL and esophageal cancer.

Supplementary Data Note

Analysis of MBD4 alterations in MSI colorectal cancer samples

A number of studies have found the BER protein *methyl-CpG binding domain 4* (*MBD4*) to be altered at high rates in MSI colorectal cancers due to MSI-induced mutations in polynucleotide tracts (8-11). As BER impairment can cause increased transition mutations at methylated CpGs (12-14), we sought to determine whether *MBD4* alteration might be the cause of the increased mutation rate at methylated cytosines that we observe in MSI colorectal cancers (see **Fig 1b**). We found four MSI colorectal cancer samples (40%) to harbour a somatic single-nucleotide thymine deletion within an A_{10} tract in exon 3 of *MBD4* (**Supp Data Table**) – a site previously identified in the literature (8). This frameshift mutation results in a truncated MBD4 protein due to the introduction of a premature stop codon, producing a protein that lacks a glycosylase domain but is still able to bind 5mC (8), including G•T mismatches (15), thus competing with unaltered MBD4 via a dominant negative effect (16). The mutations occur significantly more often in MSI than MSS colorectal cancers (MSI = 4/10 and MSS = 0/44 $P < 0.001$, Fisher's exact test). This genic region encoding *MBD4* had no copy number alterations in any mutated MSI samples (**Supp Data Table**). We also find no clear distinction between mutation-methylation associations in MSI samples with and without *MBD4* truncating mutations ($P = 0.2778$, unpaired t-test; **Supp Data Fig**). (Note that we also find a structural variant [chr12:104,373,848-104,376,577] bridging an exon in the BER protein *thymine-DNA glycosylase [TDG]* in the MBD4-truncated MSI sample TCGA-A6-6781). In summary, we are unable to propose that these alterations in base excision repair proteins solely contribute to the increased mutation rate at methylated cytosines that we have observed in MSI colorectal cancers.

Supplementary Data Table

Supplementary Data Table – Deletion mutations in *MBD4* coding exons in MSI colorectal cancer samples, together with segment mean (indicating copy number) at the locus.

TCGA[#] Sample ID	Coordinates	Reference	Alternate	Segment Mean*
TCGA-D5-6540	chr3:129,155,547	CT	C	-0.0225
TCGA-AD-A5EJ	chr3:129,155,547	CT	C	0.0211
TCGA-AA-A01R	chr3:129,155,547	CT	C	-0.0226
TCGA-A6-6781	chr3:129,155,547	CT	C	0.0094^

[#] TCGA = The Cancer Genome Atlas
* Segment mean between -0.2 and 0.2 approximates a copy number of 2
^ For primary tumour sample TCGA-A6-6781-01A; -0.025 was listed as the segment mean for primary tumour sample TCGA-A6-6781-01B

Supplementary Data Figure

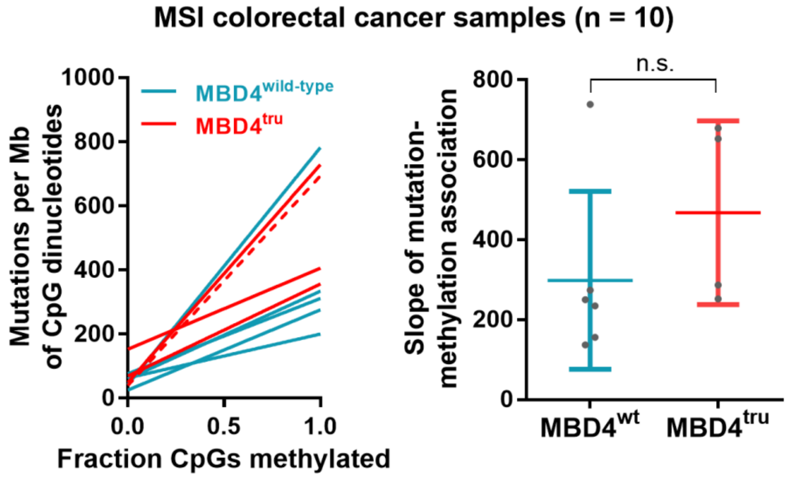

Supplementary Data Figure – Evaluation of base excision repair (BER) and mCpG mutation rate in colorectal cancers with microsatellite instability (MSI). Line of best fit from binned data of mutation-methylation associations in MSI colorectal cancers (left panel), together with a comparison of the slope of mutation-methylation association for *MBD4* wild-type and mutant samples (right panel). The sample with a structural variant in *TDG* is indicated with a dotted line. Significance is by unpaired t-test, where n.s. denotes not significant. *wt* denotes wild-type and *tru* denotes 'truncated'.

Supplementary Material References

1. Alexandrov, L.B., Nik-Zainal, S., Wedge, D.C., Aparicio, S.A.J.R., Behjati, S., Biankin, A.V., Bignell, G.R., Bolli, N., Borg, A., Borresen-Dale, A.-L. *et al.* (2013) Signatures of mutational processes in human cancer. *Nature*, **500**, 415-421.
2. Heitzer, E. and Tomlinson, I. (2014) Replicative DNA polymerase mutations in cancer. *Curr Opin Genetics Dev*, **24**, 107-113.
3. Shinbrot, E., Henninger, E.E., Weinhold, N., Covington, K.R., Göksenin, A.Y., Schultz, N., Chao, H., Doddapaneni, H., Muzny, D.M., Gibbs, R.A. *et al.* (2014) Exonuclease mutations in DNA polymerase epsilon reveal replication strand specific mutation patterns and human origins of replication. *Genome research*, **24**, 1740-1750.
4. Zheng, C.L., Wang, N.J., Chung, J., Moslehi, H., Sanborn, J.Z., Hur, J.S., Collisson, E.A., Vemula, S.S., Naujokas, A., Chiotti, K.E. *et al.* (2014) Transcription restores DNA repair to heterochromatin, determining regional mutation rates in cancer genomes. *Cell reports*, **9**, 1228-1234.
5. Roadmap Epigenomics Consortium, Kundaje, A., Meuleman, W., Ernst, J., Bilenky, M., Yen, A., Heravi-Moussavi, A., Kheradpour, P., Zhang, Z., Wang, J. *et al.* (2015) Integrative analysis of 111 reference human epigenomes. *Nature*, **518**, 317-330.
6. The Encode Project Consortium. (2012) An Integrated Encyclopedia of DNA Elements in the Human Genome. *Nature*, **489**, 57-74.
7. Ramírez, F., Dündar, F., Diehl, S., Grüning, B.A. and Manke, T. (2014) deepTools: a flexible platform for exploring deep-sequencing data. *Nucleic Acids Research*, **42**, W187-191.
8. Bader, S., Walker, M., Hendrich, B., Bird, A., Bird, C., Hooper, M. and Wyllie, A. (1999) Somatic frameshift mutations in the MBD4 gene of sporadic colon cancers with mismatch repair deficiency. *Oncogene*, **18**, 8044-8047.

9. Evertson, S., Wallin, A., Arbman, G., Rutten, S., Emterling, A., Zhang, H. and Sun, X.F. (2003) Microsatellite instability and MBD4 mutation in unselected colorectal cancer. *Anticancer research*, **23**, 3569-3574.

10. Riccio, A., Aaltonen, L.A., Godwin, A.K., Loukola, A., Percesepe, A., Salovaara, R., Masciullo, V., Genuardi, M., Paravatou-Petsotas, M., Bassi, D.E. *et al.* (1999) The DNA repair gene MBD4 (MED1) is mutated in human carcinomas with microsatellite instability. *Nat Genet*, **23**, 266-268.

11. Yamada, T., Koyama, T., Ohwada, S., Tago, K., Sakamoto, I., Yoshimura, S., Hamada, K., Takeyoshi, I. and Morishita, Y. (2002) Frameshift mutations in the MBD4/MED1 gene in primary gastric cancer with high-frequency microsatellite instability. *Cancer letters*, **181**, 115-120.

12. Wong, E., Yang, K., Kuraguchi, M., Werling, U., Avdievich, E., Fan, K., Fazzari, M., Jin, B., Brown, A.M.C., Lipkin, M. *et al.* (2002) Mbd4 inactivation increases C→T transition mutations and promotes gastrointestinal tumor formation. *Proc Natl Acad Sci U S A*, **99**, 14937-14942.

13. Millar, C.B., Guy, J., Sansom, O.J., Selfridge, J., MacDougall, E., Hendrich, B., Keightley, P.D., Bishop, S.M., Clarke, A.R. and Bird, A. (2002) Enhanced CpG Mutability and Tumorigenesis in MBD4-Deficient Mice. *Science*, **297**, 403.

14. Vasovcak, P., Krepelova, A., Menigatti, M., Puchmajerova, A., Skapa, P., Augustinakova, A., Amann, G., Wernstedt, A., Jiricny, J., Marra, G. *et al.* (2012) Unique mutational profile associated with a loss of TDG expression in the rectal cancer of a patient with a constitutional PMS2 deficiency. *DNA Repair*, **11**, 616-623.

15. Hendrich, B., Hardeland, U., Ng, H.-H., Jiricny, J. and Bird, A. (1999) The thymine glycosylase MBD4 can bind to the product of deamination at methylated CpG sites. *Nature*, **401**, 301-304.

16. Bader, S.A., Walker, M. and Harrison, D.J. (2007) A human cancer-associated truncation of MBD4 causes dominant negative impairment of DNA repair in colon cancer cells. *Br J Cancer*, **96**, 660-666.
